# BK and JC polyomavirus co-infection resulting in polyomavirus nephropathy and progressive multifocal leukoencephalopathy at the same time, a case report

**DOI:** 10.1016/j.bjid.2025.104607

**Published:** 2026-01-20

**Authors:** Sébastien Briol, Laura Labriola, Arnaud Devresse, Benoit Kabamba, Valeria Onofrj, Leïla Belkhir

**Affiliations:** a Université Catholique de Louvain, Cliniques Universitaires Saint-Luc, Department of Internal Medicine and Infectious Diseases, Brussels, Belgium; b Université Catholique de Louvain, Cliniques Universitaires Saint-Luc, Department of Nephrology, Brussels, Belgium; c Université Catholique de Louvain, Cliniques Universitaires Saint-Luc, Department of Microbiology, Brussels, Belgium; d Université Catholique de Louvain, Institute of Experimental and Clinical Research (IREC), Department of Radiology, Brussels, Belgium; e Université Catholique de Louvain, Institute of Experimental and Clinical Research (IREC), Brussels, Belgium

**Keywords:** Co-infection, JC virus, BK virus, Progressive multifocal leukoencephalopathy, Polyomavirus nephropathy

## Abstract

John Cunningham Virus (JCV) and BK Virus (BKV) are the most extensively studied Human Polyomaviruses (HPyVs), primarily associated with Progressive Multifocal Leukoencephalopathy (PML) and BKV Nephropathy (BKVN), respectively. While co-infection with these viruses has been documented in the same disease, simultaneous manifestation in distinct pathologies has not been previously reported. We present the first documented case of concurrent PML and Polyomavirus Nephropathy (PVN) due to reactivation of two different HPyVs, offering new insights into the clinical spectrum of HPyV-related diseases.

## Highlights

• First Reported Case: Simultaneous PML (JCV) and PVN (BKV) in a non-transplanted patient.
• Beyond Transplant Patients: Highlights the need to consider polyomavirus nephropathy in hematologic malignancies.
• High Viral Loads & Cross-Organ Impact: Suggests JCV may contribute to kidney injury and BKV may play a role in PML.
• Clinical Implications: Supports systematic testing for both JCV and BKV in atypical PML or PVN cases.

## Introduction

Human Polyomaviruses (HPyVs) are a ubiquitous family of 14 small, non-enveloped, double-stranded DNA viruses. HPyV seroprevalence can reach up to 99 %, with BK Virus (BKV) detected in 87.6 % and JC virus (JCV) in 55.6 % of individuals.^1^ The two best-characterized members, JCV and BKV, establish latency in urinary epithelium and can reactivate under immunosuppressive conditions. JCV reactivation is associated with Progressive Multifocal Leukoencephalopathy (PML), while BKV reactivation leads to Nephropathy (BKVN), cystitis, and ureteral strictures.^2^ HPyV infections typically occur early in life and persist asymptomatically in immunocompetent individuals, with reactivation primarily linked to impaired cellular immunity.^1^^,^^3^

## Case-report

We describe a 76-year-old man with Multiple Myeloma (MM) undergoing third-line treatment with pomalidomide and dexamethasone. He presented with a subacute decline in cognitive function, including disorientation and behavioral changes suggestive of frontal syndrome. Laboratory tests revealed stable anemia, a normal white blood cell count without inflammatory markers, normal liver function, and no electrolyte disturbances; however, acute kidney injury was noted (with a creatinine increasing from 1.64 mg/dL to 3.19 mg/dL in less than two months).

**Fig. 1:**
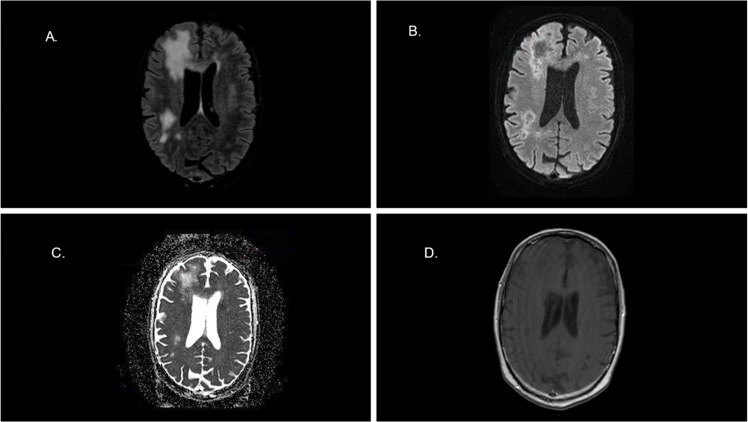
(A) 3D-FLAIR image shows areas of hyperintense signal in the white matter of the right hemisphere involving the U fibers. (B) DWI and respective ADC map (C) show that lesions manifest a hypersignal wavefront spreading without diffusion restriction (lack of hyposignal in ADC map). This finding indicates an activee myelinoclastic process at the wavefront. (D) The axial T1 image after contrast medium administration shows no enhancement of the lesions.

A cerebral CT scan demonstrated multifocal white matter lesions, raising suspicion of PML. Subsequent brain MRI revealed multiple supratentorial myelinoclastic lesions consistent with PML. Cerebrospinal Fluid (CSF) analysis showed no pleocytosis, normal glucose, protein, and lactate levels, a negative bacterial culture, and a JC virus PCR result of 150,236 copies/mL. PCR was performed using the JCV ELITe MGB® and BKV ELITe MGB® kits on the ELITe BeGenius analytical platform from ELITech, with respective limits of quantification in blood and CSF of 306 copies/mL and 239 copies/mL for JCV; 165 copies/mL and 250 copies/mL for BKV (Fig. 1 and 2).

Evaluation of kidney function showed unremarkable urinary sediment and renal ultrasound findings. Urinalysis revealed no electrolyte imbalances or albuminuria but showed an elevated beta-2-microglobulin at > 4000 mg/L. Given the context of polyomavirus reactivation, further testing confirmed BKV infection, with positive urinary PCR, decoy cells, and positive immunostaining. Additionally, BK viremia was markedly elevated (Table 1) which was consistent with the diagnosis of presumptive BKVN. PCR testing for JCV in blood and BKV in CSF also returned positive (Table 1), confirming the diagnosis of simultaneous JCV- and BKV-associated disease.

**Table 1:** PCR results in blood; CSF and urine with 2 different assays specific for each virus.

	Blood	CSF	Urine
JC virus	130,573 cop/mL	150,236 cop/mL	/
BK virus	> 100,000,000 cop/mL	696 cop/mL	> 2500,000 cop/mL
	> 170,000,000 IU/mL	907 IU/mL	> 4250,000 IU/mL

Despite discontinuation of immunosuppressive therapy, the patient’s neurological and renal function continued to decline. Respecting the patient’s will of comfort, he was transferred to palliative care.

## Discussion

PML is a rare Central Nervous System (CNS) disease primarily caused by JCV. It occurs predominantly in patients with Human Immunodeficiency Virus (HIV)/Acquired Immunodeficiency Syndrome (AIDS), (approximately 80 % of cases) but is also associated with immunosuppression due to hematological malignancies (10 %), multiple sclerosis (5 %), and organ transplantation or chronic inflammatory/rheumatological diseases, notably with treatments such as natalizumab (10 %).^3^ PML has been reported in both remission and relapsed MM, with a high mortality rate (a serie of reports by Koutsavlis et al. revealed a mortality of 10/16 patients).^4^ Diagnosis requires either direct identification of JCV in brain tissue with characteristic histopathology or a combination of progressive neurological symptoms, MRI findings typical of PML, and a positive JCV PCR in CSF.^5^ Mortality varies by patient population, with a one-year survival rate of 60 % in HIV patients and 77 % in natalizumab-associated PML, whereas hematological malignancy-associated PML has a 90 % mortality rate within two months.^3^ Although not covered in recent reviews, notably by Cortese et al.,^3^ rare case reports describe PML caused by BKV.

Polyomavirus-associated Nephropathy (PVN), primarily due to BKV reactivation, affects 1 %–10 % of Kidney Transplant Recipients (KTR), with graft loss occurring in 30 %–80 % of cases. A plasma BK viral load >10,000 copies/mL is associated with BKVN, while levels > 1000,000 copies/mL predicts extensive BKVN.^6^ A definitive diagnosis of BKVN requires a kidney biopsy, but a presumptive diagnosis can be made when viral load exceeds 10,000 copies/Ml. .^6^^,^^7^ While literature on BKVN in native kidneys is limited, cases have been reported, particularly in patients with hematologic malignancies.^2^ BKV is also implicated in hemorrhagic cystitis and ureteral stenosis, primarily in hematopoietic stem cell transplant recipients and, less frequently, in KTRs.^6^

Detection of JCV or SV40 (another polyomavirus) in PVN is rare. JCV is responsible for fewer than 3 % of PVN cases in KTRs.^8^^,^^9^ Although less extensively studied, JCV viruria and viremia appear to contribute to PVN less frequently than BKV viremia and may present later than BK in the post-transplantation follow-up.^9^ In a review of native kidney disease, only one case demonstrated JCV co-activation in the renal medulla.^2^ Another review of KTRs found JCV co-infection in 44 of 140 BKVN cases, though none of these patients developed PML. Notably, concurrent JCV viremia was linked to poorer renal outcomes.^10^ However, the relationship between JCV and BKV remains debated, with some studies suggesting a potential negative interaction between the two viruses.^9^ JCV-associated PVN has been reported concurrently with PML, even though caution is advised with nonspecific PCR used in some studies, which detects both viruses indiscriminately. A small study conducted at our institution^11^ did not detect any JCV-associated PVN.

Management of both BKVN and PML primarily involves reduction of immunosuppression. In our patient, immunosuppression was due to both the underlying disease and its treatment. While large studies do not establish a strong association between MM therapies and PML,^4^ immune reconstitution was not achievable in this case, leading to a rapid clinical decline.

This case report has several key implications. First, it is the first documented instance of simultaneous BKV and JCV reactivation leading to concurrent PVN and PML in a non-transplanted patient. The only comparable reports describe a case of BKVN in a native kidney later progressing to PML[2] and a case of PVN (unspecified) in a KTR followed by JCV-related PML.^12^ Another KTR case described co-infection with JCV and BKV resulting in ureteral lesions.^13^ Additionally, two cases of PML were suspected to involve BK and JC co-infection.^14^^,^^15^

Second, the PCR documentation in this case raises the hypothesis that high JCV viremia may have contributed to progressive nephropathy, while BKV could have played a role in PML. However, definitive confirmation would have required histopathological evidence.

Lastly, this case underscores the need to investigate BKVN beyond KTRs, including in patients with hematologic malignancies, hematopoietic cell transplants, non-renal organ transplants, and HIV.^2^ The insights gained from this report highlight the potential interplay between JCV and BKV in nephropathy as well as in PML, suggesting that polyomavirus co-reactivation should be systematically explored when diagnosing polyomavirus-associated diseases.

The main limitation of this report is the absence of histopathological analysis. However, the PCR findings from blood, CSF, and urine provide strong evidence for the involvement of both viruses, particularly given that high-level BK viremia is a well-established predictor of nephropathy.

In conclusion, this case provides valuable new insights in the understanding of polyomaviruses-associated pathologies as well as the interactions of the members of this family leading to organ damage.

## Data availability

The data that support the findings of this study are available from the corresponding author upon reasonable request.

## Conflicts of interest

The authors declare no conflicts of interest.
